# Retinal Protection of New Nutraceutical Formulation

**DOI:** 10.3390/pharmaceutics17010073

**Published:** 2025-01-07

**Authors:** Luca Rosario La Rosa, Veronica Pepe, Francesca Lazzara, Giovanni Luca Romano, Federica Conti, Erika Giuffrida, Claudio Bucolo, Santa Viola, Giuseppe De Pasquale, Maria Cristina Curatolo, Cristina Zappulla

**Affiliations:** 1 Innovation and Medical Science, SIFI S.p.A., 95025 Aci Sant’Antonio, Italy; pepe.veronica@sifigroup.com (V.P.); santa.viola@sifigroup.com (S.V.); giuseppe.depasquale@sifigroup.com (G.D.P.); cristina.curatolo@sifigroup.com (M.C.C.); cristina.zappulla@sifigroup.com (C.Z.); 2 Department of Biomedical and Biotechnological Sciences, School of Medicine, University of Catania, 95123 Catania, Italy; francesca.lazzara@unict.it (F.L.); federica.conti@unict.it (F.C.); erika.giuffri@gmail.com (E.G.); claudio.bucolo@unict.it (C.B.); 3 Center for Research in Ocular Pharmacology–CERFO, University of Catania, 95125 Catania, Italy; giovanniluca.romano@unikore.it; 4 Department of Medicine and Surgery, “Kore” University of Enna, 94100 Enna, Italy

**Keywords:** retinal function, RGC degeneration, neuroprotection, dietary supplement, glaucoma, citicoline, homotaurine, epigallocatechin-3-gallate, forskolin

## Abstract

**Background/Objectives:** Retinal ganglion cell (RGC) protection represents an unmet need in glaucoma. This study assessed the neuroprotective, antioxidant, and anti-inflammatory effect of a new nutraceutical formulation named Epicolin, based on citicoline, homotaurine, epigallocatechin-3-gallate, forskolin, and vitamins, through in vitro and in vivo studies. **Methods:** The neuroprotective effect of Epicolin or its single components, and Epicolin compared to an untreated control and two marketed formulations [Formulation G (FG) and N (FN)], was evaluated in neuroblastoma cells (SH-SY5Y) challenged with staurosporine. The antioxidant potential and the scavenging activity of Epicolin compared to the untreated control, and FG and FN, was evaluated in SH-SY5Y cells and through oxygen radical absorbance capacity acellular assay, respectively. Moreover, the protective effect against hypoxic damage was evaluated in Muller cells (MIO-M1) subjected to hypoxia. The efficacy of Epicolin was also evaluated in DBA/2J glaucomatous mice through the use of a pattern electroretinogram (PERG), immunostaining, and real-time PCR. **Results:** Among the nutraceutical formulations tested, only Epicolin showed a significant neuroprotective effect on SH-SY5Y attributable to the synergistic action of its single ingredients. As for antioxidant and scavenging activity, Epicolin showed a higher efficacy compared to FG and FN. Furthermore, Epicolin showed the same protective effect on MIO-M1 cells reducing HIF-1α expression. Finally, Epicolin treatment on DBA/2J mice protected the RGCs from loss of function, as demonstrated by PERG analysis, and attenuated their death by enhancing brain-derived neurotrophic factor (*BDNF*) and reducing interleukin-1 beta (*IL-1β*) and tumor necrosis factor-alpha (*TNF-α*) expression. **Conclusions:** Epicolin, due to its neuroprotective, antioxidant, and anti-inflammatory properties, represents a promising potential treatment for glaucoma.

## 1. Introduction

Glaucoma is a complex neurodegenerative disorder marked by optic neuropathy, visual field loss, and retinal degeneration, characterized by the apoptosis of retinal ganglion cells (RGCs), ultimately leading to vision loss. It is the leading cause of irreversible blindness and is projected by the World Health Organization to affect over 112 million people by 2040 [1,2]. Although its exact pathophysiology remains to be fully elucidated, it is well established that neurotrophic factor deficiency and inflammation are likely major contributing factors [3]. Elevated intraocular pressure (IOP) is recognized as the primary risk factor, yet despite the effectiveness of IOP-lowering treatments, the disease continues to progress in a significant proportion of patients [4].

A hallmark of glaucoma is progressive damage to axons and the loss of RGCs [2,5], which can occur even before noticeable increases in IOP occur [6]. RGC death results from a cascade of pathological events, inflammation, and also the production of reactive oxygen species (ROS), oxidative stress, and glutamate-induced neurotoxicity [7]. Additionally, elevated IOP can lead to compromised local blood flow and reduced oxygen availability (hypoxia) in the retina, subjecting cells to severe stress and protein damage. This triggers the activation of hypoxia-inducible factors (HIFs), which facilitate metabolic adaptation to hypoxia by increasing glycolysis through the upregulation of glucose transporters 1 (GLUT1) and 3 (GLUT3) [8,9]. HIFs mediate a cascade of transcriptional activation, primarily in Müller cells and astrocytes [10]. These factors, specifically HIF-1α, can also upregulate the expression of nicotinamide adenine dinucleotide phosphate oxidase type 2 (NOX-2) and inducible nitric oxide synthase (iNOS), leading to the production of reactive oxygen species (ROS). This ROS generation can, in turn, stimulate further HIF-1α expression, establishing a feedback loop that amplifies inflammation through glial activation and apoptosis [11].

Nutrient and oxygen deficiencies impair RGCs and also significantly increase ROS production, leading to oxidative stress [8] that can cause RGC death by inhibiting key enzymes involved in the tricarboxylic acid cycle, the mitochondrial electron transport chain, and calcium homeostasis, resulting in defective energy metabolism. Elevated oxidative stress markers have been detected in the aqueous humor of patients with both primary open-angle glaucoma (POAG) and primary closed-angle glaucoma (PACG) [12].

Therefore, in light of all these premises, the development of neuroprotective strategies, whether independent of or in addition to IOP-lowering drugs, represents an unmet medical need and remains a critical challenge. Recent advancements have identified neuroprotective molecules that can prevent visual field loss and preserve visual function.

The most effective neuroprotective strategy involves addressing the primary neurodegenerative mechanisms to slow the progression of glaucoma. This approach encompasses three key strategies: neuroprotection, neuroregeneration, and neuroenhancement. Neuroprotection focuses on slowing or preventing neuronal death to maintain physiological functions. Neuroregeneration aims to promote neuronal regeneration and restore connections between the eye and the brain in damaged axons. Neuroenhancement seeks to improve the function of surviving but impaired RGCs [13]. Further, the combination of retinal protection and decreased IOP has been explored in animal models [14].

In addition to pharmacological treatments, there is growing evidence supporting the efficacy of nutritional supplements in managing retinal ganglion cell degeneration associated with glaucoma. Nutritional supplementation has been shown to reduce IOP, enhance optic nerve circulation, modulate excitotoxicity, and promote RGC survival [15,16,17,18]. Combining multiple active molecules that target different mechanisms may offer a more robust approach to preventing RGC degeneration compared to single-agent treatments.

On this premise, a new nutraceutical supplement, covered by a patent and named Epicolin, has been developed as an “all in one” well-balanced formulation intended to be used in cases of glaucoma in association with pharmacology standard therapy and as preventative therapy in people at risk of developing glaucoma.

Specifically, this new dietary supplement was designed to combine the action of several natural substances well known in the literature for their neuroprotective and antioxidant effect and IOP-lowering effects, such as citicoline [2,4,12,19], forskolin from Coleus forskohlii [7,18], homotaurine [13,17], epigallocatechin-3-gallate (EGCG) from green tea leaves extract [20,21,22,23,24], and vitamins, which are important cofactors in the metabolic processes necessary for human health and energy metabolism. Moreover, some of the ingredients are also endowed with antioxidant potential, such as thiamine (vitamin B1) [25,26], riboflavin (vitamin B2) [13,26,27,28], nicotinamide (vitamin B3) [2,11,29,30], pyridoxine (vitamin B6) [13,25,31], folic acid (vitamin B9) [26,32], and α-tocopherol (vitamin E) [33,34].

Based on this premise, the primary objective of the present study was to investigate, in vitro, the neuroprotective effects of Epicolin formulation in comparison to its individual components to assess the potential synergistic effect of the single components when mixed together. Subsequently, the neuroprotective and antioxidant effects of Epicolin formulation were evaluated in vitro and compared to other nutraceutical formulations currently available on the market, hereafter referred to as Formulation G and Formulation N. Moreover, the effect of the formulations was evaluated in an in vitro model of glaucoma by using Muller cells (MIO-M1) and hypoxic challenge. Finally, the neuroprotective and anti-inflammatory properties of Epicolin formulation were examined in an in vivo mouse model of glaucoma.

## 2. Materials and Methods

### 2.1. Materials

SH-SY5Y human neuroblastoma cell lines (ATCC-CRL-2266) were obtained from LGC Standards S.r.l. (Milan, Italy). Nutrient mixture F-12 Ham (F-12), minimum essential medium eagle (MEM), Fetal Bovine Serum (FBS), penicillin-streptomycin, Fluorescein sodium salt, MEM non-essential amino acid solution 100× (NEAA), streptomycin, cobalamin (vitamin B12), glucose oxidase (GOx), phosphate-buffer saline (PBS), 2,2′-Azobis(2-amidinopropane) dihydrochloride (AAPH), 6-hydroxy-2,5,7,8-tetramethylchroman-2-carboxylic acid (Trolox), 3-(4,5-dimethylthiazol-2-yl)-2,5-diphenyltetrazolium bromide tetrazolium (MTT), staurosporine (STS), RIPA buffer, and a protease and phosphatase inhibitor cocktail were from Sigma-Aldrich (Milan, Italy). L-glutamine was purchased from Lonza (Basel, Switzerland).

Human Moorfields/Institute of Ophthalmology-Müller 1 (MIO-M1) was purchased from the Institute of Ophthalmology, University College of London (UCL, London, UK). GlutaMAX^TM^ DMEM medium was purchased from Gibco, ThermoFisher (Waltham, MA, USA). Petri dishes were purchased from Costar, Corning (New York, NY, USA).

Citicoline base, homotaurine, green tea leaf extract (titrated at 95% of epigallocatechin-3-gallate, EGCG), Coleus forskohlii (titrated at 10% of forskolin), thiamine (vitamin B1), riboflavin (vitamin B2), nicotinamide (vitamin B3), pyridoxine (vitamin B6), folic acid (vitamin B9) and α-tocopherol (vitamin E), sunflower seed oil, sunflower lecithin, and geleol glyceryl stearate were obtained from Chemo Iberica S.A., part of Insud Pharma S.L.U. (Madrid, Spain). Peppermint extract was purchased from Sergio Fontana s.r.l. (Canosa di Puglia, BT, Italy). Dimethyl sulfoxide (DMSO) was obtained from Merck Life Science s.r.l. (Milan, Italy). A lactate dehydrogenase (LDH) cytotoxicity assay kit was purchased from Cayman Chemical (Ann Arbor, MI, USA). Nutilis was purchased from Nutricia (Hoofddorp, The Netherlands). Primary antibody for HIF-1α was purchased from Novus Biologicals (Mouse, CO, USA), and that for actin from Sigma-Aldrich (Rabbit, St. Louis, MI, USA). Secondary chemiluminescent antibodies (ECL anti-mouse and ECL anti-rabbit) were purchased from Cytiva Amersham (Amersham, UK). A BCA Assay Kit (Pierce™ BCA Protein Assay Kit) was purchased rfom Invitrogen, Life Technologies, Carlsbad, CA, USA. NuPAGETM 10% Bis-Tris gel was purchased from ThermoFisher, Waltham, MA, USA. Nitrocellulose membrane was purchased from Bio-Rad, Hercules, CA, USA. ECL (SuperSignal™ West Pico PLUS Chemiluminescent Substrate) was purchased from Thermo Fisher Scientific, Carlsbad, CA, USA.

Primary antibody for RNA binding protein, mRNA Processing Factor (RBPMS, RNA-binding protein with multiple splicing), was purchased from Novus Biologicals, part of Bio-Techne SRL (Milan, Italy). Secondary goat anti-rabbit antibody was obtained from Abcam (Cambridge, UK).

Sucrose, paraformaldehyde, and Triton X-100 were purchased from Sigma-Aldrich (St. Louis, MO, USA). RNase-free water was obtained from Ambion (Austin, TX, USA). Fluoromount-G (DAPI), TRIzol reagent, SuperScript II Reverse Transcriptase, primers for the analysis of *interleukin-1 beta* (*IL-1β*), *18S ribosomal RNA* (*18S*), and *brain-derived neurotrophic factor* (*BDNF*) genes by quantitative real-time PCR were purchased from Eurofin Genomics (Milan, Italy). QuantiNova SYBR Green Real-Time PCR Kit and *tumor necrosis factor-alpha* (*TNF-α*) primers were purchased from Qiagen (Milan, Italy).

Tiletamine + zolazepam (60 mg/kg, Zoletil) were purchased from Virbac (Milan, Italy). Medetomidine (40 μg/kg, Domitor) was obtained from Orion Pharma s.r.l. (Milan, Italy).

### 2.2. Cells and Culture Conditions

Human neuroblastoma cells, SH-SY5Y, were grown in a complete culture medium composed of F-12 and MEM in a 1:1 ratio, supplemented with 15% FBS, 100 U/mL penicillin and 100 µg/mL streptomycin, and 2 mM of L-glutamine and NEAA. The cells were kept in a humidified 5% CO_2_ atmosphere at 37 °C.

When the confluence reached 80%, the cells were counted and seeded at 2 × 10^5^ cells per well in a 96-well plate. Three days after seeding, the experiment was performed.

Human Moorfields/Institute of Ophthalmology-Müller 1 (MIO-M1) was cultured at 37 °C (humidified atmosphere with 5% CO_2_) in GlutaMAX^TM^ DMEM medium with 100 U/mL penicillin, 100 μg/mL streptomycin, and 10% FBS. After reaching confluence (∼70%), MIO-M1 was used for experimental procedures.

### 2.3. Treatments Used in the In Vitro Cellular and Acellular Studies

The active ingredients tested and their concentrations in the in vitro protocols were as follows:

The Epicolin formulation contained citicoline base (52.1 μg/mL), homotaurine (17.7 μg/mL), green tea leaf extract (EGCG, 27 μg/mL), Coleus forskohlii (68 μg/mL), and vitamins B1 (0.96 μg/mL), B2 (4.1 μg/mL), B3 (4.76 μg/mL), B6 (0.59 μg/mL), B9 (0.16 μg/mL), and E (17.1 μg/mL). The ingredients listed above were tested individually or mixed together in formulation, i.e., as Epicolin.

Formulation G (FG) contained homotaurine (25.5 μg/mL), peppermint extract (153 μg/mL), coleus forskholii (34 μg/mL), and vitamins B1 (0.54 μg/mL), B2 (0.72 μg/mL), B3 (4.77 μg/mL), B6 (0.72 μg/mL), and B12 (0.88 ng/mL).

Formulation N (FN) contained citicoline base (177.5 μg/mL), homotaurine (17.7 μg/mL), and vitamin E (8.17 μg/mL).

All single ingredients were prepared as a stock solution in DMSO, with the exception of citicoline base, homotaurine, vitamins B5 and B8, and zinc, dissolved in water. The stock solutions were sterilized by filtration. Finally, working solutions were obtained by properly dissolving the stock solutions in complete culture medium without FBS.

### 2.4. In Vitro Neuroprotective Activity on SH-SY5Y Cells

Sub-confluent SH-SY5Y cells were pre-treated for 6 h with Epicolin formulation or its single ingredients as specified in Section 2.3. Moreover, three other formulations were also tested: i.e., FG and FN. The cells were then challenged with STS 250 nM for a further 18 h to induce apoptosis as already described, with some modification, by Barrachina et al. [35]. Cells stimulated with STS, without any treatment but containing the same DMSO concentration as the treatments, were used as the control (CTRL).

Viability was evaluated after 24 h (6 h + 18 h), replacing the medium in each well with 100 µL of MTT, 0.4 mg/mL, in complete culture medium. Following 30 min of incubation, MTT formazan was extracted with 100 µL of 100% DMSO. The optical density (O.D.) of samples was obtained by reading at 570 nm using a microplate reader (Infinite 200 Pro M-Plex, Tecan, Männedorf, Switzerland). All of the samples were tested in triplicate for at least three different experimental days.

Moreover, to assess the synergistic effect of the components when in a mixture (Epicolin), the Bliss method [36,37,38] was used to calculate the combination index (*CI*). The *CI* indicates synergy, antagonism, or additivity when it is less than, greater than, or equal to 1, respectively [39]. Briefly, all effects observed in the in vitro neuroprotective assay and expressed as a percentage with respect to the control (CTRL) were normalized to the maximum (*E_Max_*) and minimum (*E_Min_*) effect values, as described in Supplementary Table S1, so as to be expressed in the form of probability (0 ≤ *E* ≤ 1), as required by the Bliss approach [40]:

(1) $$E_{N_{i}}=\frac{E_{i}-E_{Min}}{{E_{Max}-E}_{Min}}$$

The *CI* is calculated as follows (see Supplementary Table S1):

(2) $$CI=\frac{E_{e}}{E_{o}},$$

where *E_e_* is the expected effect and *E_o_* is the normalized observed effect of the formulation. The expected effect *E_e_* is determined using the following formula:

(3) $$1-\prod_{i=1}^{n}(1-E_{N_{i}})$$

where *E_Ni_* represents the normalized effect of each individual formulation, while *E_o_* corresponds to the observed effect of the formulation Epicolin (see Supplementary Table S1).

### 2.5. In Vitro Antioxidant Activity on SH-SY5Y Cells

SH-SY5Y cells were pre-treated for 6 h with the following formulations: Epicolin, FG, or FN. After six hours of incubation, the cells were challenged for 18 h with 5.3 mU/mL of GOx to exert oxidative insult by H_2_O_2_ production [41,42,43]. Cells stimulated with GOx, without any treatment but containing the same DMSO concentration as the treatments, were used as the CTRL. Cytotoxicity resulting from cell incubation with the treatments was determined by LDH assay performed on the culture medium. After the treatment time had elapsed (6 h + 18 h), 100 µL of the supernatant was taken and distributed in a new 96-well plate together with 100 µL of LDH solution prepared using the commercial LDH assay kit. After an incubation period of 30 min at 37 °C, the O.D. was read at 490 nm (Infinite 200 Pro M-Plex, Tecan, Männedorf, Switzerland).

The percentage cell viability values were then obtained with the following formula:

100 − [(O.D. − O.D._SPONT_)/(O.D._MAX_ − O.D._SPONT_) × 100] (4)

where O.D. indicates the absorbance of the sample under examination, while O.D._SPONT_ and O.D._MAX_ indicate the corresponding absorbance linked to the spontaneous and maximum release of LDH in the culture medium and represents 0 and 100% cytotoxicity, respectively. All samples were tested in triplicate for at least three different experimental days.

### 2.6. Antioxidant Scavenging Activity Assay

The acellular in vitro oxygen radical absorbance capacity (ORAC) assay was used as previously described with some modification [44,45]. Briefly, 25 μL of each formulation (i.e., Epicolin, FG or FN) was added to 150 μL sodium fluorescein 4 μM in PBS and incubated for 15 min at 37 °C. Then, 25 μL of the peroxyl radical generator AAPH (0.41% in PBS 75 mM) was added, and the decay of fluorescein was measured at its maximum emission of 520 nm (excitation at 480 nm) every hour for 20 h using a Tecan Infinite 200 Pro M-Plex microplate reader (Männedorf, Switzerland). The area under the curve (AUC) was calculated for each sample, subtracting the AUC of the blank. The results were then expressed as the μM trolox equivalent (TE) using a trolox calibration curve. All of the samples were tested in triplicate on at least three different experimental days.

### 2.7. Hypoxia Damage Protocol and Western Blot Analysis

MI0-M1 was exposed to low percentage of oxygen (~1%) by means of a hypoxia incubator chamber (Stemcell technologies, Vancouver, BC, Canada) to induce hypoxia damage. Particularly, 1.5 × 10^4^ or 6 × 10^5^ cells were seeded in 96-well plates or 60 mm Petri dishes (Costar, Corning, New York, NY, USA) for cell viability (MTT) and Western blot analysis, respectively. After 48 h of culture, the cells were pre-treated with Epicolin or FG or FN for 2 h under normoxic conditions, and then placed in the hypoxia incubator chamber for 4 h.

MTT assay and Western blot were carried out. As regards normoxia, the cells were treated with Epicolin, FG, or FN for 6 h, and then an MTT assay was carried out (see Supplementary Figure S1).

Medium without any treatment (CTRL) and medium without any treatment but with DMSO (CTRL + DMSO) were used as controls in both the normoxia and hypoxia conditions.

After hypoxia exposure, the cells were subjected to an MTT assay for cell viability and Western blot analysis for the evaluation of HIF-1α protein levels. MIO-M1 cells cultured under normoxic conditions were used as the control. Proteins from cell lysates were extracted with RIPA Buffer, including a protease and phosphatase inhibitor cocktail. The total protein content in each cell lysate sample was determined using the BCA Assay Kit. Extracted proteins (20 µg) were loaded on the NuPAGE TM 10% Bis-Tris gel. After electrophoresis, the proteins were transferred into a nitrocellulose membrane. The membranes were blocked with milk 5% in Tris-buffered saline 0.2% Tween 20 (TBST) for 1 h at room temperature. Then, the membranes were incubated overnight (4 °C) with the appropriate primary anti- HIF-1α (1:500 dilution) and anti- actin (Rabbit, 1:1000 dilution) antibodies. After overnight incubation, the membranes were then incubated with secondary chemiluminescent antibodies (1:1000 dilution) for 1 h at room temperature. After secondary antibodies were added, the membranes were incubated with ECL and were detected through I-BrightTM 1500 by chemiluminescence. Densitometry analyses of blots were performed at non-saturating exposures and analyzed using ImageJ software 1.53 (NIH, Bethesda, MD, USA). The values were normalized to actin, which was used as a housekeeping control.

### 2.8. In Vivo Model of Glaucoma, DBA/2J Mice

DBA/2J female mice [46] from Jackson Laboratories (Bar Harbor, ME, USA), six months in age, were randomly assigned to either the Epicolin treatment (Epicolin, n = 16) or the vehicle-treated group (CTRL, n = 16). All procedures were performed in compliance with the Directive 2010/63/UE European Convention for the Protection of Vertebrate Animals used for Experimental and Other Scientific Purposes and to the Association for Research in Vision and Ophthalmology (ARVO) statement for the use of animals in ophthalmic and vision Research. The experimental protocol was approved by the Italian Ministry of Health (authorization no. n. 885/2021-PR of 09/11/2021). The animals were housed under standard conditions, with free access to standard food and water, in a light-controlled (12 h light/12 h dark) room with standard temperature and humidity conditions (21 ± 3 °C and 54 ± 4% humidity).

The animals were orally treated once a day with 208.6 mg/kg of Epicolin active components and excipients (i.e., sunflower seed oil; sunflower lecithin, and geleol glyceryl stearate) suspended in food thickener, Nutilis. The selected Epicolin dose was obtained similarly to the human dose (one cap per day) and in increasing concentrations considering the different metabolism of the mice [47]. In particular, 200 µL of each suspension was administered by oral gavage (20-gauge needle) to each mouse, once per day for 5 weeks. The control animals were treated with Nutilis (vehicle).

**Figure 1:**
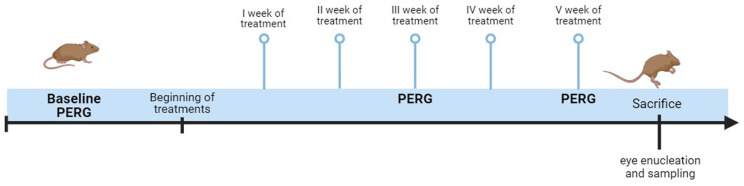
Experimental design. Baseline pattern electroretinogram (PERG) recording was performed before treatment. Epicolin treatment was carried out daily for 5 weeks. PERG recordings were carried out after three and five weeks of treatment. At the end of the 5 weeks, the mice were sacrificed and their tissues processed for immunohistochemical staining and molecular biology analyses.

RGC function was evaluated by means of pattern electroretinogram (PERG) before the beginning of the treatment (baseline, week 0) and after 3 and 5 weeks of treatment (Figure 1). Moreover, at the end of the experiments, i.e., 5 weeks, the mice were sacrificed and their retinas collected for immunohistochemical staining and molecular biology analyses as described below.

### 2.9. Pattern Electroretinogram in DBA/2J Mice

PERG recordings were carried out to assess RGC function at week 0 and after 3 and 5 weeks of treatment. As previously described [15,48], the mice were anesthetized by tiletamine + zolazepam (60 mg/kg) and medetomidine (40 μg/kg) and transferred onto a heating plate with the superior incisor teeth hooked to a bite bar; the head was gently restrained with head holders. The body was kept at a constant temperature (37.0 °C) with a feedback-controlled heating pad (TCAT-2LV, Physitemp Instruments, Inc., Clifton, NJ, USA). A small drop of balanced saline was topically applied to prevent corneal dryness during PERG recordings. The PERG was recorded simultaneously for each eye using a commercially available instrument (Jorvec Corp., Miami, FL, USA) through a single subcutaneous electrode positioned in the snout. Visual stimuli consisted of black and white horizontal bars (pattern) generated on LED tablets, which were presented independently to each eye at a 10 cm distance (56° vertical × 63° horizontal field; spatial frequency, 0.05 cycles/degree; 98% contrast; 800 cd/m^2^ mean luminance; right-eye reversal, 0.992 Hz; left-eye reversal, 0.984 Hz).

Electrical signals recorded from the common snout electrode were averaged (>1110 epochs), and PERG responses from each eye were isolated by averaging at stimulus-specific synchrony. PERG waveforms consisted of a positive wave (defined as P1) followed by a slower negative wave with a broad trough (defined as N2), both automatically detected using Jorvec software 2.0. Therefore, each waveform was analyzed by measuring the peak-to-trough (P1-N2) amplitude, which is defined as PERG amplitude; meanwhile, the time-to-peak of the P1 wave is defined as PERG latency [49]. The PERG analysis, regarding the left and right eye, was performed independently because the development of glaucoma in DBA/2J mice is asymmetrical [50], the same as in humans [51].

### 2.10. Immunostaining and Immunofluorescence Analyses in DBA/2J Mouse Retinas

The mice were sacrificed by cervical dislocation and their eyeballs were enucleated. Twenty-four eye globes (n = 12 CTRL and n = 12 Epicolin) were fixed in 4% *w*/*v* paraformaldehyde in PBS 0.1 M pH 7.4 for 2 h at room temperature. Then, the eye globes were washed with PBS and stored at 4 °C in 30% *w*/*v* PBS sucrose solution. Each retina was isolated, rinsed with PBS, and incubated with primary antibodies diluted in PBS containing 2% *v*/*v* triton X-100 and 5% *v*/*v* FBS. The RBPMS primary antibody was used for retinal immunohistochemistry, with a dilution of 1:500. After 48 h of incubation with primary antibodies at 4 °C, the retinas were washed with PBS and incubated with the goat anti-rabbit secondary antibodies (1:200) for 24 h at 4 °C. Finally, the retinas were rinsed with PBS once again, flat mounted on polarized glass slides, and cover slipped in a fluorescence mounting medium with DAPI (Fluoromount-G). Images were acquired by using a Zeiss Observer Z1 microscope (Carl Zeiss Microscopy GmbH, Oberkochen, Germany). Four radial opposite tiles were analyzed in both the central (500 μm away from the optic nerve head) and the peripheral (500 μm away from the peripheral edge) portions of each retina. RGC densities were calculated, respectively, as the number of RBPMS-positive cells normalized to the scanned area, using ImageJ software 1.53 (ImageJ software, NIH, Bethesda, MD, USA).

### 2.11. Real-Time PCR in DBA/2J Mice Retinas

The mice were sacrificed by cervical dislocation and their eyeballs were enucleated. Twenty retinas per experimental group, i.e., CTRL and Epicolin, were used for qPCR analyses; in particular, retinas were collected from each eye, then two retinas were pooled in a vial and homogenized (n = 10 independent retinal samples per experimental group). The total RNA was extracted, purified, and suspended in RNase-free water using TRIzol reagent. The A_260_/A_280_ ratio of the optical density of RNA samples (measured with Nanodrop spectrophotometer ND-1000, Thermofisher) was 1.95–2.01, confirming RNA purity. cDNA was synthesized from 1 μg RNA with a SuperScript™II reverse transcription kit. Quantitative real-time PCR (RT-PCR) was performed with the Rotor-Gene Q using Qiagen QuantiNova SYBR Green Real-Time PCR Kit. The amplification reaction mix included 1 μL (100 ng) of cDNA. Forty-five amplification cycles were carried out for each sample, performed in triplicate. Melting curve analysis confirmed the specificity of the amplified products. The results were analyzed with the 2^−ΔΔCt^ method and expressed as fold change vs. control. The analyzed genes were normalized to *18S* mRNA levels, a constitutively expressed gene encoding for ribosomal RNA. The qPCR analyses adhered to the MIQE guidelines [52]. The primer sequences are listed in Table 1.

### 2.12. Statistical Analysis

Graphs were created and statistical analysis was performed with GraphPad Prism 6 and 10 (GraphPad Software, La Jolla, CA, USA).

The data generated by all experiments are reported as means ± SEMs. One sample *t*-test versus 50% cut-off [53] was performed to evaluate the cytotoxicity effects of the treatments on MIO-M1 cells.

One-way analysis of variance (ANOVA) was carried out followed by the appropriate post hoc test for multiple comparisons: Dunnett’s post hoc test for in vitro cellular assays and Sidak’s post hoc test for antioxidant scavenging activity and hypoxic damage. Tukey’s post hoc test was used for the analysis of PERG in vivo data and MIO-M1 cytotoxicity assay. Finally, for immunostaining and real-time PCR, a *t*-test was performed. Differences between the groups were considered statistically significant for *p*-values ≤ 0.05.

## 3. Results

### 3.1. In Vitro Neuroprotective Activity on Neuroblastoma Cells

**Figure 2:**
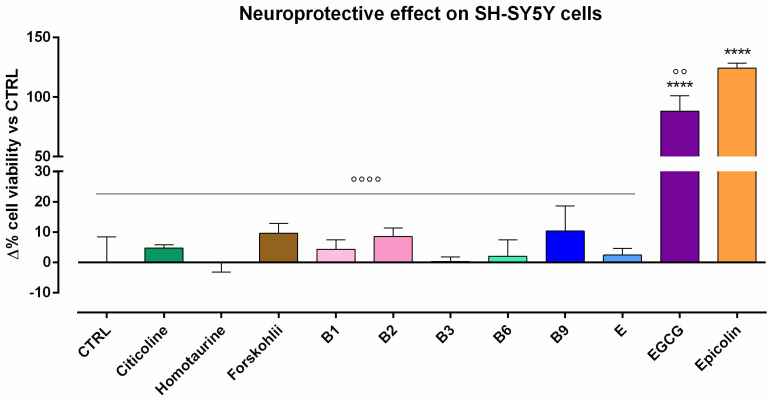
Neuroprotection on SH-SY5Y cells induced with staurosporine (STS, 250 nM) and treated with Epicolin formulation or its single ingredients. The data are represented as percentage differences (Δ) compared to the control. The data represent means ± SEMs of at least three different experimental days in triplicate. Statistical analysis was performed using one-way ANOVA plus Dunnett’s post hoc test. **** *p* ≤ 0.0001 vs. control (CTRL); °° *p* ≤ 0.01; °°°° *p* ≤ 0.0001 vs. Epicolin.

The neuroprotective effect of single ingredients and their associations, i.e., Epicolin formulation, were investigated on the SH-SY5Y cell line. To this end, in vitro neurotoxicity was induced by STS and the neuroprotective effect, in terms of cell viability, was evaluated by MTT assay. The results demonstrated that, among single components, only EGCG was endowed with a statistically significant neuroprotective effect with respect to the CTRL (**** *p* ≤ 0.0001, Figure 2). Importantly, all single ingredients when mixed together, i.e., Epicolin formulation, showed a higher protective effect (**** *p* ≤ 0.0001 vs. CTRL), even compared to EGCG-treated cells (°° *p* ≤ 0.01) (Figure 2).

Moreover, to assess whether a synergistic interaction occurs when the individual components are combined in the Epicolin formulation, the combination index (*CI*) was calculated, yielding a value of 0.796, thereby confirming the presence of a synergistic effect of the single ingredients when mixed together, i.e., Epicolin (see Supplementary Table S1).

**Figure 3:**
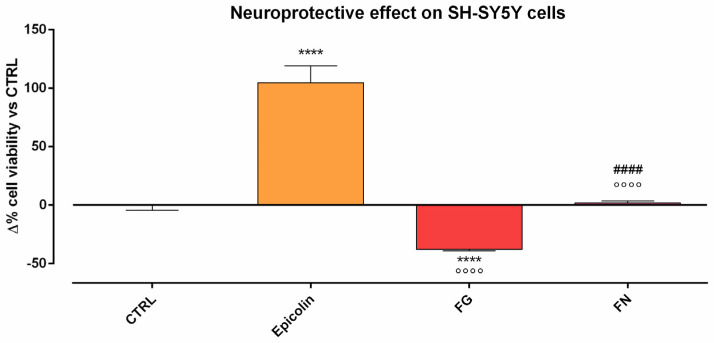
Neuroprotection on SH-SY5Y cells induced with staurosporine (STS, 250 nM) and treated with Epicolin, Formulation G (FG), or Formulation N (FN). The data are represented as percentage differences (Δ) compared to the control (CTRL). The data represent means ± SEMs of at least three different experimental days in triplicate. Statistical analysis was performed using one-way ANOVA plus Dunnett’s post hoc test. **** *p* ≤ 0.0001 vs. control (CTRL); °°°° *p* ≤ 0.0001 vs. Epicolin; #### *p* ≤ 0.0001 vs. FG.

Further experiments were performed to compare the neuroprotective effect of Epicolin formulation with other marketed nutraceutical supplements, i.e., FG and FN. Epicolin formulation exerted a statistically protective effect on SH-SY5Y cells after STS exposure, compared to CTRL (**** *p* ≤ 0.0001, Figure 3). Furthermore, FG and FN failed to protect SH-SY5Y cells from staurosporine-induced death, FN did not appear to differ from CTRL, and FG treatment resulted in a statistically significant but even greater staurosporine-induced cell death compared to the CTRL condition (**** *p* ≤ 0.0001; Figure 3) and that of FN (#### *p* ≤ 0.0001, Figure 3). Importantly, all other tested formulations (i.e., FG and FN) did not show a protective effect compared to Epicolin (°°°° *p* ≤ 0.0001, Figure 3).

### 3.2. In Vitro Antioxidant Activity on Neuroblastoma Cells

**Figure 4:**
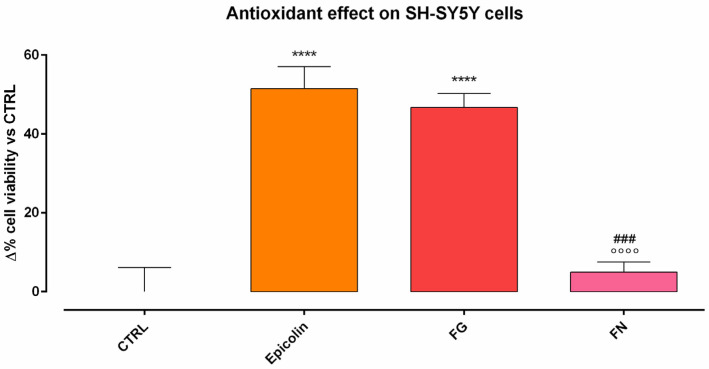
Antioxidant effect on SH-SY5Y cells induced with glucose oxidase enzyme (GOx, 5.3 mU/mL) and treated with Epicolin, Formulation G (FG), or Formulation N (FN). The data are represented as percentage differences (Δ) compared to the control (CTRL). The data represent the means ± SEMs of at least three different experimental days in triplicate. Statistical analysis was performed using one-way ANOVA plus Dunnett’s post hoc test. **** *p* ≤ 0.0001 vs. CTRL; °°°° *p* ≤ 0.0001 vs. Epicolin; ### *p* ≤ 0.001 vs. FG.

The antioxidant effect of Epicolin formulation was evaluated on the SH-SY5Y cell line in comparison with other marketed nutraceutical supplements, i.e., FG and FN. To this end, the oxidative stress was induced by the GOx enzyme, and the antioxidant effect, in terms of cell viability, was evaluated by LDH assay. The results showed that only Epicolin and FG exerted a statistically significant protective effect on SH-SY5Y cells against GOx-mediated oxidative activity, both compared to the CTRL (**** *p* ≤ 0.001; Figure 4). Importantly, FN was statistically different with respect to Epicolin (°°°° *p* ≤ 0.0001, Figure 4) and FG (### *p* ≤ 0.001, Figure 4), while no difference was found in terms of efficacy between Epicolin and FG.

### 3.3. Antioxidant Scavenging Activity Assay

**Figure 5:**
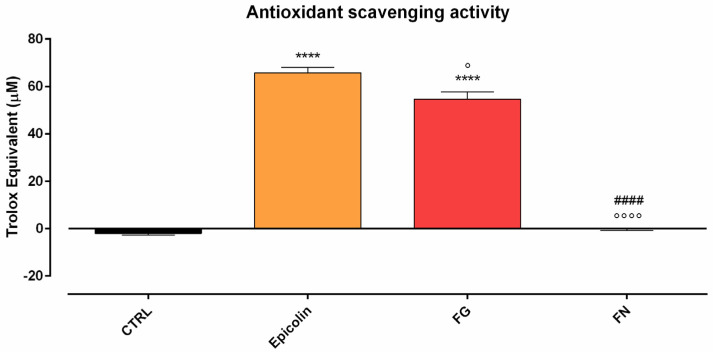
Scavenging antioxidant activity of Epicolin compared to Formulation G (FG) or Formulation N (FN). The data are represented as the trolox equivalent (µM). The data represent the means ± SEMs of at least three different experimental days in triplicate. Statistical analysis was performed using one-way ANOVA plus Sidak’s post hoc test. **** *p* ≤ 0.0001 vs. control (CTRL); ° *p* ≤ 0.05, °°°° *p* ≤ 0.0001 vs. Epicolin; #### *p* ≤ 0.0001 vs. FG.

The antioxidant scavenging activity of the Epicolin formulation was evaluated in an acellular assay in comparison with other marketed nutraceutical supplements, i.e., FG and FN. To this end, the antioxidant scavenging activity against AAPH was evaluated by ORAC assay. The results demonstrated that Epicolin and FG exerted a statistically significant antioxidant scavenging activity against AAPH-mediated fluorescein oxidation compared to CTRL (**** *p* ≤ 0.001, Figure 5), while FN did not. Importantly, FN was statistically different with respect to Epicolin (°°°° *p* ≤ 0.0001, Figure 5) and FG (#### *p* ≤ 0.0001, Figure 5), and there was a significant difference in terms of efficacy between FG and Epicolin. In fact, Epicolin showed the highest efficacy in terms of antioxidant scavenging activity (° *p* ≤ 0.05, Figure 5) in vitro.

### 3.4. In Vitro Protective Effect Against Hypoxic Damage

The cell viability (MTT) assay showed no cytotoxicity for treatments on MIO-M1 cells in normoxic conditions; they appear, in fact, statistically different from the 50% cut-off for cytotoxicity (## *p* ≤ 0.01, ### *p* ≤ 0.001, #### *p* ≤ 0.0001; see Supplementary Figure S1a). In addition, Epicolin treatment appeared to be the only tested treatment statistically different to normoxic CTRL + DMSO (++ *p* ≤ 0.01; see Supplementary Figure S1a) and to hypoxic CTRL + DMSO (**** *p* ≤ 0.0001; see Supplementary Figure S1b). Moreover, Epicolin showed a viability that was statistically different from FG (°° *p* ≤ 0.01; see Supplementary Figure S1b) and FN (°°° *p* ≤ 0.001; see Supplementary Figure S1b).

**Figure 6:**
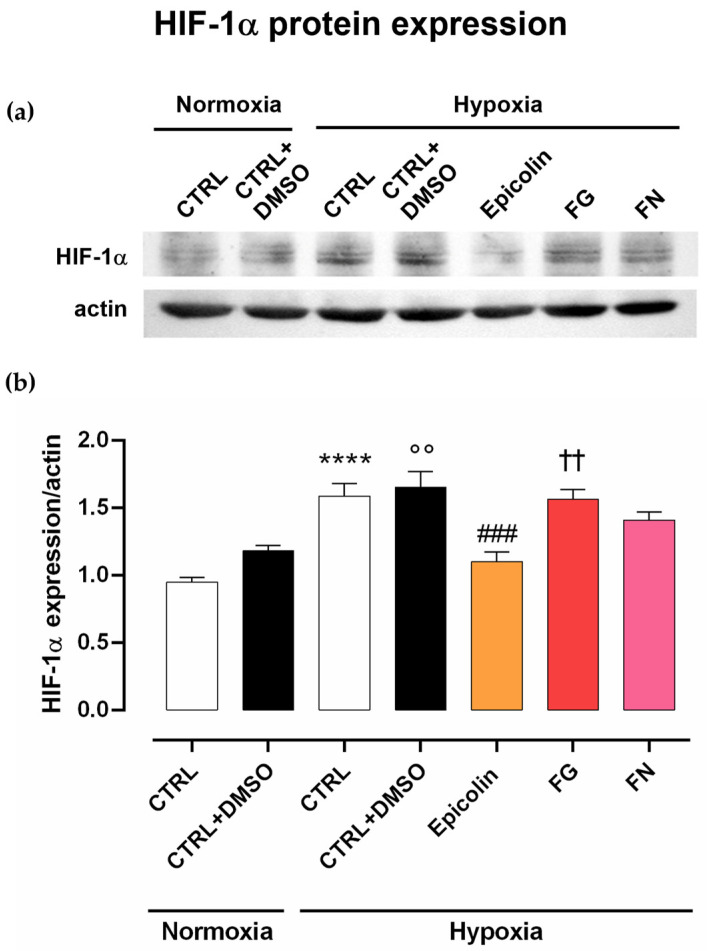
Effect of Epicolin, Formulation G (FG), and Formulation N (FN) on hypoxia-inducible factors (HIF-1α) protein levels. Human Moorfields/Institute of Ophthalmology-Müller 1 (MIO-M1) cells were pre-treated in normoxic conditions for 2 h with formulations and then exposed to hypoxia insult for 4 h. (**a**) Representative immunoblots and (**b**) densitometric analysis of HIF-1α in lysates of MIO-M1. Densitometry analysis of each band was carried out with the Image J program. HIF-1α expression has been normalized to actin values. The values are reported as the mean ± SEM of n = 4. The data were analyzed using one-way ANOVA and Sidak’s post hoc test for multiple comparisons. **** *p* ≤ 0.0001 vs. CTRL (normoxia); °° *p* ≤ 0.01 vs. CTRL + DMSO (normoxia); ### *p* ≤ 0.001 vs. CTRL + DMSO (hypoxia); †† *p* ≤ 0.01 vs. Epicolin.

As shown in Figure 6, four hours of hypoxia challenge led to a significant increase in HIF-1α expression in control samples (hypoxia, CTRL, and CTRL + DMSO) compared to normoxic control conditions (**** *p* ≤ 0.0001 vs. normoxia CTRL; °° *p* ≤ 0.01 vs. normoxia CTRL + DMSO), whereas Epicolin significantly reduced HIF-1α expression, compared to CTRL + DMSO, in hypoxic condition (### *p* ≤ 0.001, Figure 6). Moreover, Epicolin showed a protective effect against hypoxia insult through a stronger inhibition of HIF-1α expression, compared to FG (†† *p* ≤ 0.01) and FN, although the difference from the latter was not statistically significant (Figure 6). Finally, FN and FG treatments did not significantly reduce the hypoxia-induced increase in HIF-1α expression in MIO-M1 cells compared to CTRL + DMSO in hypoxic conditions.

### 3.5. Retinal Protection in DBA/2J Mice

**Figure 7:**
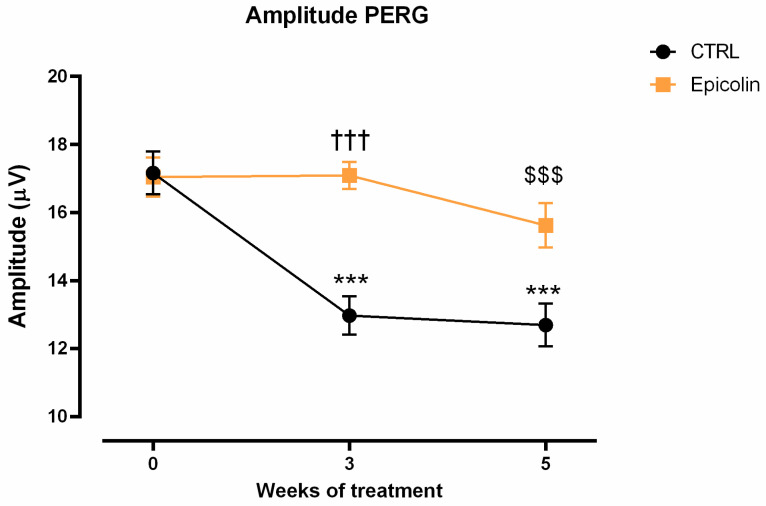
Evaluation of retinal ganglion cell (RGC) function with pattern electroretinogram (PERG) (amplitude values vs. time of treatment). PERG was recorded at baseline (week 0), and at the 3rd and 5th week of treatment. The data were plotted as means ± SEMs (n = 16 mice per group). *** *p* ≤ 0.001 vs. week 0; ††† *p* ≤ 0.001 vs. the control (CTRL) at 3 weeks; $$$ *p* ≤ 0.001 vs. CTRL at 5 weeks. Two-way ANOVA plus Tukey’s post hoc test.

Retinal function was assessed by PERG analysis in DBA/2J mice, of six months of age, orally treated by gavage once a day with Nutilis (CTRL) or Epicolin formulation (208.6 mg/kg). Analysis was carried out before the start of the treatment, i.e., week 0 and after 3 and 5 weeks of treatment. According to the established progression of retinal dysfunction in DBA/2J mice, PERG amplitude significantly decreased in the CTRL group after 3 and 5 weeks of treatment. (*** *p* ≤ 0.001, Figure 7). Nevertheless, Epicolin formulation was able to slow down the RGC loss function; indeed, amplitude values after both 3 (††† *p* ≤ 0.001 vs. CTRL 3 weeks) and 5 weeks of treatment ($$$ *p* ≤ 0.001 vs. CTRL 5 weeks) were significantly higher than that of the CTRL group (Figure 7).

### 3.6. Immunostaining and Immunofluorescence Analyses on DBA/2J Mice Retinas

**Figure 8:**
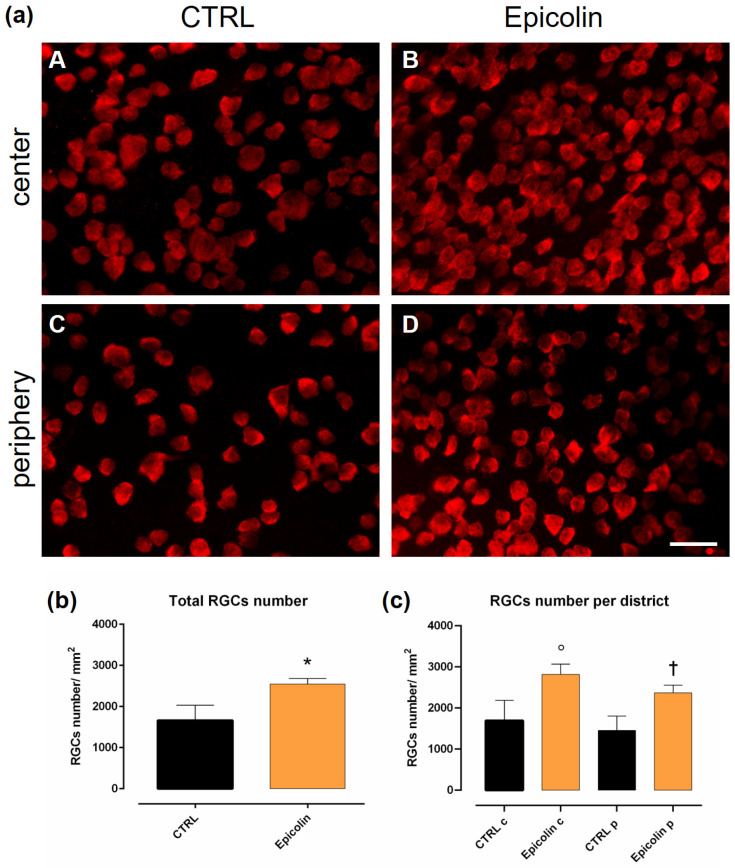
Immunostaining of RNA-binding protein and mRNA Processing Factor-positive (RBPMS+) cells for the analysis of retinal ganglion cell (RGC) density in flat-mount retinas. (**a**) RBPMS+ RGCs in central (**A**,**B**) and peripheral areas (**C**,**D**) of control (CTRL) and Epicolin formulation-treated mice. Quantification was carried out on the whole retina (**b**) and on both central and peripheral areas of the retina (**c**). Scale bar corresponds to 50 μm. The data were plotted as means ± SEMs (n = 12 retinas per group). * *p* ≤ 0.05 vs. CTRL; ° *p* ≤ 0.05 vs. CTRL c; † *p* ≤ 0.05 vs. CTRL p. Unpaired *t*-test. CTRL central (CTRL c), CTRL periphery (CTRL p), Epicolin central (Epicolin c), and Epicolin periphery (Epicolin p).

Quantitative analysis of RGCs was carried out by immunostaining for RBPMS (RNA-binding protein with multiple splicing), widely used for the analysis of general RGC population density [54]. The analysis was performed in DBA/2J mice retinas after 5 weeks of oral treatment (once daily) with Nutilis (CTRL) or Epicolin formulation (208.6 mg/kg) in order to analyze the potential protective effect of Epicolin in reducing the loss of RGCs compared to glaucomatous mice. The anatomical evaluation reflects the functional assessment carried out by way of PERG; indeed, the average density of RGCs was significantly (* *p* ≤ 0.05) higher in the Epicolin-treated mice group compared to the CTRL group, both in the periphery (Epicolin p) and center (Epicolin c) of the retinas (Figure 8a–c).

### 3.7. Gene Expression by Real-Time PCR in DBA/2J Mice Retinas

**Figure 9:**
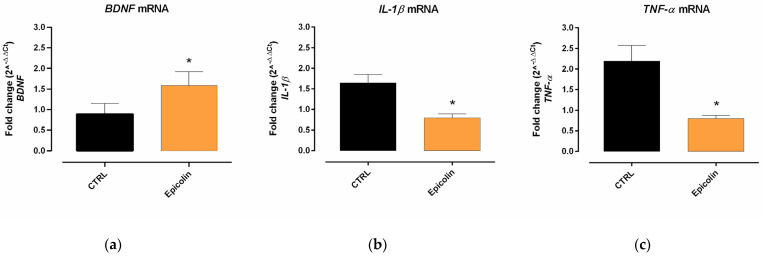
Epicolin formulation effect on neurotrophic and inflammatory biomarkers. (**a**) Brain-derived neurotrophic factor (*BDNF*), (**b**) interleukin-1 beta (*IL-1β*), and (**c**) tumor necrosis factor-alpha (*TNF-α*) expression of Epicolin-treated mice compared to the control (CTRL) group. Each bar represents the mean value ± SEM (n = 10 per group). * *p* ≤ 0.05 vs. CTRL. Unpaired *t*-tests were performed.

To assess the neuroprotective and anti-inflammatory effect of Epicolin formulation (208.6 mg/kg) in glaucomatous mice after 5 weeks of oral treatment (once a day), the mRNA expression of *BDNF* and inflammatory markers such as *IL-1β* and *TNF-α* was analyzed. The results showed that Epicolin formulation was able to significantly exert a neuroprotective effect, increasing the *BDNF* mRNA levels with respect to CTRL mice (* *p* ≤ 0.05, Figure 9a). Furthermore, the mRNA levels of *IL-1β* (Figure 9b) and *TNF-α* (Figure 9c) were significantly downregulated in Epicolin-treated mice compared to the CTRL mice (glaucomatous retinas), thus showing an anti-inflammatory effect.

## 4. Discussion

Glaucoma is a complex neurodegenerative disorder with mechanisms that remain only partially understood. It leads to vision loss primarily through the degeneration and death of RGCs, which play a pivotal role in the process of vision. Apoptosis is widely regarded as the primary mechanism of RGC death in glaucoma. This process can be initiated by various signals, originating from the cell soma, the axons, or from external signals in the extracellular environment [55,56]. The current therapeutic strategies approved for glaucoma focus on lowering IOP, a well-established risk factor for the disease [55]. However, approximately one-third of patients with POAG exhibit optic nerve cupping and visual field defects without any elevation in IOP, a condition known as normotensive glaucoma (NTG). While hypotensive medications have shown some mild beneficial effects in patients with NTG, the degeneration of RGCs in glaucoma has been found to persist in both POAG and NTG, showing that other factors can induce RGC death and irreversible vision loss. Recently, accumulating evidence has highlighted the role of neuroinflammation, ischemia/hypoxia, mitochondrial dysfunction, oxidative stress, and innate immune responses in the development and progression of glaucoma despite IOP [16,55], especially in the case of prolonged inflammatory response, which can represent a high risk factor for RGC survival [57].

The use, in addition to drug therapy, of various natural substances, as single ingredients or in combination, was found to be effective in counteracting retinal neurodegeneration in diseased eyes [12,18,58,59,60,61].

Thus, the aim of the present study was to demonstrate, through both in vitro and in vivo models, the neuroprotective and antioxidant activity of a new nutraceutical formulation, Epicolin, composed of citicoline, homotaurine, coleus forskohlii, green tea leaf extract, B group vitamins, and vitamin E.

The SH-SY5Y cell line, derived from human neuroblastoma, is widely used as a neuron-like cell in vitro model for studying neuronal biology due to being particularly sensitive to oxidative stress and neurotoxic insults, making it relevant for modeling pathological conditions of the retina. While the RGC-5 cell line has been previously used a RGC model, significant doubts about its origin have raised concerns regarding its reliability and validity as an RGC model [62,63]. Given these uncertainties, the present study used the SH-SY5Y cell line as a more generalized neuronal-like in vitro model for neurotoxicity and neuroprotection and validated the findings in a more specific in vivo model of glaucoma to ensure the translational relevance and robustness of the results.

Firstly, the neuroprotective effect of single ingredients and their association, i.e., Epicolin formulation, was investigated on the SH-SY5Y cell line to assess whether the combination in a mixture could increase the overall efficacy or, conversely, reduce it, using a neuroprotection model already known in the literature for testing the activity of citicoline [35]. The results highlight how the neuroprotective activity of the Epicolin formulation was higher than that of the single ingredients. Such a result suggests that this effect may depend on a synergistic interaction among its different components. This speculation is supported by data in the literature documenting cases of synergy for natural compounds [18,64] contained in Epicolin formulation and supports the evidence that no single ingredient, by itself, can have a neuroprotective effect comparable to that of the entire formulation. Importantly, preliminary studies on SH-SY5Y cells demonstrated the cytocompatibility of the individual components or their mixtures, i.e., Epicolin formulation, at the tested concentration [65,66].

The results of the neuroprotective data highlight how the activity of Epicolin is mainly mediated by the effect of EGCG, which is well known in the literature for its neuroprotective activity and in glaucoma [20,21,22,23,24,67], but the addition of the other Epicolin constituents enhances this effect in a statistically significant manner.

Once the usefulness of mixing the individual components together has been proven, subsequent studies was focused exclusively on Epicolin formulation to assess its functional gain in comparison with other formulations available on the market and to provide a rationale for advancing its evaluation in other study models.

In the second instance, using the same in vitro model, the neuroprotective effect of the Epicolin formulation was evaluated in comparison with other formulations, i.e., FG and FN, which represent, in terms of composition and proportion among constituting ingredients, other dietary supplements already on the market. The results demonstrated that Epicolin was a unique formulation able to counteract staurosporine-induced cell death and highlighted its potential in protecting RGCs from apoptosis, the main cause of RGC loss in glaucoma.

Glaucomatous pathology is characterized by increased oxidative stress induced by the production of free radicals, which play a key role in the damage and death of RGCs by inhibiting key enzymes in energy metabolism [12]. For this reason, the Epicolin formulation was further evaluated by assessing its antioxidant potential, also in comparison with FG and FN mixtures. Such antioxidant activity was evaluated both in an in vitro cellular model of antioxidant activity and in an acellular model of antioxidant scavenging activity; the latter related exclusively to the intrinsic chemical nature of the test item, independent of specific cellular mechanisms. The results of the in vitro model of cellular antioxidant activity showed that only the Epicolin and FG formulations were able to counteract the oxidative effect mediated by the H_2_O_2_ produced by GOx, while the FN formulation failed to do so, showing no difference compared to the CTRL. Finally, the results for antioxidant scavenging activity confirmed the data of the in vitro cellular antioxidant assay, but Epicolin significantly increased the antioxidant scavenging effect. Taken together, the results of the in vitro tests showed that, among the formulations tested, Epicolin was the only one capable of combining both neuroprotective and antioxidant activity, pivotal targets of glaucoma.

Moreover, in this study, the effect of Epicolin formulation compared to FG and FN on Müller cells (MIO-M1) has been investigated. Müller glia have multiple functions and are symbiotically associated with RGCs. Indeed, glial dysfunctions contribute to glaucoma onset [68]. In particular, it has been demonstrated that in DBA/2J mice, Müller cells become reactive at a very early stage, before RGCs damage [69]. This means that Müller cell dysfunctions play a key role in the pathogenesis of glaucoma, exerting a neurotoxic effect on RGCs, leading to neurodegeneration [70].

The results demonstrated that the Epicolin formulation showed the best effect in reducing HIF-1α proteins in an in vitro model of hypoxia compared to FG and FN. The reduction in the hypoxic pathway activation in Müller cells could lead to a reduced cytotoxic effect of retinal glia on RGCs by the inhibition of HIF-1α-induced inflammation and oxidative stress.

Based on the promising results obtained in vitro, and considering that the efficacy of Epicolin compared with the two marketed formulations, i.e., FG and FN, was evaluated using two different cell lines (neuroblastoma cells and Müller cells) and across four distinct in vitro models (neuroprotection, oxidative stress, antioxidant scavenging activity, and hypoxia), in alignment with the principles of the 3Rs (Replacement, Reduction, and Refinement) and our commitment to minimizing animal use, we decided to proceed with an in vivo study in a mouse model of glaucoma to evaluate the efficacy of the Epicolin formulation compared only with the vehicle. Including FG and FN in the in vivo experiments would have significantly increased the number of animals used without adding proportionate value, given the already in vitro data supporting Epicolin’s potential.

DBA/2J mice are a well-established model of glaucoma characterized by the age-related progressive degeneration of retinal ganglion cells [46], starting at around 6 months of age, with a higher prevalence in females [29,71]. Specifically, the in vivo study was designed to assess whether the Epicolin formulation could counteract the progression of retinal degeneration in glaucomatous mice (DBA/2J) during the early stages of RGC degeneration. For these reasons, in vivo treatment started at 6 months of age in DBA/2J mice, and the PERG, the gold standard tool for the early detection of RGC dysfunction [18], was used to measure RGC activity. The results demonstrated that Epicolin was able to reduce the progression rate of glaucoma by reducing the RGC degeneration, as evidenced by higher PERG amplitude values in Epicolin-treated mice during the 5 weeks of treatment. Along with retinal function evaluation, immunohistochemical studies and molecular biology analyses were carried out. According to the PERG results, immunohistochemistry demonstrated that the number of RGCs was preserved by Epicolin treatment; indeed, the number of RNA-binding proteins with multiple splicing (RBPMS)-positive cells was higher than in untreated glaucomatous mice. In addition, molecular biology analyses showed that Epicolin significantly upregulated the neurotrophic factor *BDNF*, which is known for its neuroprotective effect [72] and is significantly reduced in glaucoma. Finally, Epicolin treatment was able to counteract inflammatory mediator expression, which is increased in older DBA/2J mice. In fact, the mRNA levels of two pro-inflammatory markers, *TNF-α* and *IL-1β*, were significantly downregulated in the Epicolin-treated mouse group compared to the control group.

These findings have significant translational implications, indicating that Epicolin can substantially influence the expression of proteins and genes associated with the pathogenesis of glaucoma. This supports the potential use of such a nutritional supplement in glaucoma patients, particularly in the early stages of the disease.

## 5. Conclusions

Overall, the present study demonstrates that the Epicolin formulation, compared to the other formulations tested in vitro, is able to combine in a unique formulation a neuroprotective and antioxidant effect. Moreover, in vivo data confirm the above mentioned in vitro evidence, supporting the neuroprotective profile in glaucomatous mice by preserving retinal tissue.

Epicolin, due to its combination of ingredients, may be beneficial in managing the onset and progression of glaucoma by addressing several identified causes of progressive RGCs loss, including neurotoxicity, oxidative stress, hypoxia, free radical formation, and inflammation.

The neuroprotective, antioxidant, and anti-inflammatory effects of Epicolin encourage its use in the management of glaucoma beyond the standard of care of lowering intraocular pressure. Clinical trials could be valuable to further support the efficacy of Epicolin food supplements in glaucoma management as already demonstrated in this nonclinical investigation.

## 6. Patents

Patents resulting from the work reported in this manuscript are as follows: La Rosa Luca Rosario, Pepe Veronica, and Zappulla Cristina Maria Concetta, “Nutraceutical composition with neuroprotective effect for the treatment of glaucoma”, publication number EP4356906A8·2024-07-03.

## Figures and Tables

**Table 1:** Primers used for real-time polymerase chain reaction (PCR) amplification.

Gene	Sequence
*m18S*	F: 5′-GTTCCGACCATAAACGATGCC-3′; R: 5′-TGGTGGTGCCCTTCCGTCAAT-3′
*mBDNF*	F: 5′-GTTCGAGAGGTCTGACGACG-3′; R: 5′-AGTCCGCGTCCTTATGGTTT-3′
*mTNF-α*	Mm_Tnf_1_SG; QT00104006
*mIL-1β*	F: 5′-ACATCAGCACCTCACAAGCAGAG-3′; R: 5′-TGGGGAAGGCATTAGAAACAGTC-3′

## Data Availability

Further inquiries can be directed to the corresponding authors.

## Supplementary Materials

The following supporting information can be downloaded at: https://www.mdpi.com/article/10.3390/pharmaceutics17010073/s1, Table S1. Combination index (*CI*) calculation for single ingredients when mixed in the Epicolin formulation; Figure S1. Citotoxicity assay on Human Moorfields/Institute of Ophthalmology-Müller 1 (MIO-M1) treated with Epicolin, Formulation G (FG) and Formulation N (FN) in normoxic and hypoxic conditions;
