# Supplementary material for: Retinal Protection of New Nutraceutical Formulation
Source: Pharmaceutics. 2025 Jan 7;17(1):73. doi: 10.3390/pharmaceutics17010073 (PMC11769253; doi:10.3390/pharmaceutics17010073)
Supplement: Supplementary file 1 [file pharmaceutics-17-00073-s001.zip › pharmaceutics-3363543-supplementary.pdf]

pharmaceutics

Supplementary Materials

Retinal Protection of New Nutraceutical Formulation

Luca Rosario La Rosa [1,*], Veronica Pepe [1], Francesca Lazzara [2], Giovanni Luca Romano [3,4], Federica Conti [2], Erika Giuffrida [2], Claudio Bucolo [2,3], Santa Viola [1], Giuseppe De Pasquale [1], Maria Cristina Curatolo [1] and Cristina Zappulla [1]

[1] Innovation and Medical Science, SIFI S.p.A., 95025 Aci Sant'Antonio, Italy; pepe.veronica@sifigroup.com (V.P.); santa.viola@sifigroup.com (S.V.); giuseppe.depasquale@sifigroup.com (G.D.P.); cristina.curatolo@sifigroup.com (M.C.C.); cristina.zappulla@sifigroup.com (C.Z.)
[2] Department of Biomedical and Biotechnological Sciences, School of Medicine, University of Catania, 95123 Catania, Italy; francesca.lazzara@unict.it (F.L.); federica.conti@unict.it (F.C.); erika.giuffri@gmail.com (E.G.); claudio.bucolo@unict.it (C.B.)
[3] Center for Research in Ocular Pharmacology–CERFO, University of Catania, 95125 Catania, Italy; giovanniluca.romano@unikore.it
[4] Department of Medicine and Surgery, "Kore" University of Enna, 94100 Enna, Italy
* Correspondence: luca.larosa@sifigroup.com

Data reported as supplementary material:

- **Table S1.** Combination index (*CI*) calculation for single ingredients when mixed in the Epicolin formulation.
- **Figure S1.** 3-(4,5-dimethylthiazol-2-yl)-2,5-diphenyltetrazolium bromide tetrazolium **(**MTT) assay on Human Moorfields/Institute of Ophthamology-Müller 1 (MIO-M1) treated with Epicolin, Formulation G (FG) and Formulation N (FN) in normoxic and hypoxic conditions.

Table S1. Combination index (*CI*) calculation for single ingredients when mixed in the Epicolin formulation. (a) All effects observed in the in vitro neuprotection assay were reported as percentage with respect to control (CTRL). Each value (E_i) was normalized to the maximum (E_{Max}) and minimum (E_{Min}) effect values, as described in **(b)**, to obtain the normalized effect (E_{Ni}). The expected effect (E_e) of the mixture of the single ingredients was calculated as detailed in **(c)** and used to calculate the *CI*, as outlined in **(d)**, where the observed effect (E_o) represents the normalized effect of Epicolin.

(a)

	**% Mean *vs* CTRL (E_i)**	**Δ% Mean *vs* CTRL ***	**Normalized value (E_{Ni}) (0 - 1)**
**Citicoline**	104,680	4,680	0,038
**Homotaurine**	99,983	-0,017	0,000
**EGCG**	188,181	88,181	0,710
**Forskohlii**	109,573	9,573	0,077
**Vit. B1**	104,294	4,294	0,035
**Vit. B2**	108,489	8,489	0,068
**Vit. B3**	100,282	0,282	0,002
**Vit. B6**	101,952	1,952	0,016
**Vit. B9**	110,308	10,308	0,083
**Vit. E**	102,378	2,378	0,019
**Epicolin**	224,212	124,212	1,000

** values as shown in the Figure 2 of the main text of the paper*

(b) $E_{N_i} = \frac{E_i - E_{Min}}{E_{Max} - E_{Min}}$

(c) $E_e = 1 - \prod_{i=1}^{n}(1 - E_{N_i})$

(d) $CI = \frac{E_e}{E_o}$

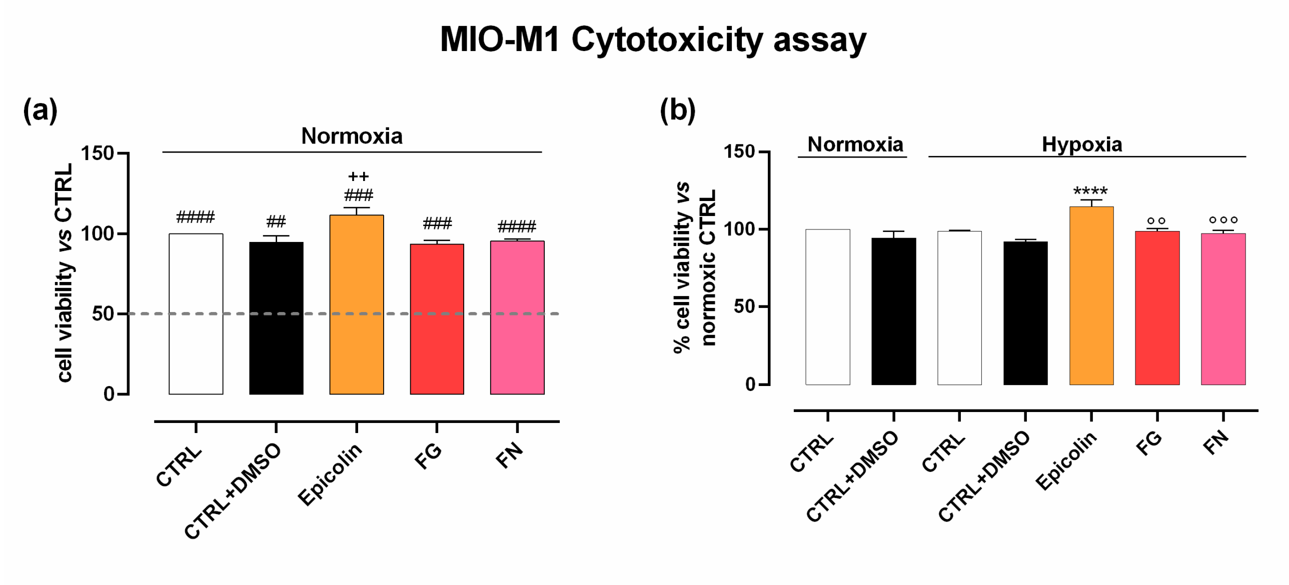

Figure S1. Citotoxicity assay on Human Moorfields/Institute of Ophthamology-Müller 1 (MIO-M1) treated with Epicolin, Formulation G (FG) and Formulation N (FN) in normoxic and hypoxic conditions. **(a)** MIO-M1 cells were treated with Epicolin, FG and FN formulations for 6h and then MTT assay was carried out. **(b)** MIO-M1 cells were pre-treated in normoxic condition for 2 hours with Epicolin, FG and FN and then exposed to hypoxia damage for 4 hours and then MTT was carried out. Data are represented as percentage compared to normoxic condition control (CTRL). Values are reported as mean ± SEM of n = 4. Statistical analysis was performed by One-way ANOVA plus Tukey's post-hoc test to compare treatment groups and by One sample t-test to compare treatment groups with the 50% cut-off.
$p \leq 0.01$, ### $p \leq 0.001$, #### $p \leq 0.0001$ vs 50% cut-off; **** $p \leq 0.0001$ vs hypoxic CTRL+DMSO; ++ $p \leq 0.01$ vs normoxic CTRL+DMSO, °° $p \leq 0.01$, °°° $p \leq 0.001$ vs Epicolin.
